# Does the serial position effect identify people at‐risk for dementia? Findings from the Canadian Longitudinal Study on Aging

**DOI:** 10.1002/alz70857_104400

**Published:** 2025-12-25

**Authors:** Sofia Marinou, Natalie A. Phillips

**Affiliations:** ^1^ Concordia University, Montreal, QC, Canada

## Abstract

**Background:**

Subjective cognitive decline (SCD) is defined as a self‐perceived decline in cognition without objective cognitive impairment and is considered a potential predictor of Alzheimer's disease (AD). However, summary‐level neuropsychological test scores often fail to detect subtle cognitive changes in this group, making assessments challenging. A promising approach is to examine the serial position effect (SPE) where, during verbal recall, items presented at the beginning (primacy) and end (recency) of a list are remembered better than ones in the middle. Primacy recall is associated with long‐term memory, whereas recency recall reflects working memory. Primacy recall may be sensitive to early changes in memory function associated with reduced hippocampal volume, so poor primacy recall in individuals with SCD could be predictive of future cognitive decline.

**Method:**

Data were taken from the comprehensive cohort (*n* = 30,097) of the Canadian Longitudinal Study on Aging (CLSA), a 20‐year national study following 50,000 Canadians aged 45–85 at baseline. To investigate whether SPE patterns differ by SCD status, participants were categorized into three groups based on a self‐report measure of SCD: no SCD, SCD+worry, and SCD‐no worry. SPE patterns were assessed using the Rey Auditory Verbal Learning Test (RAVLT).

**Result:**

All analyses controlled for sex, age, and education. There was no significant effect of SCD status on immediate recall total scores, *F*(2,15166)=0.59, *p* = 0.56, η^2^< .001. However, significant differences were observed for primacy, *F*(2,15166)=7.20, *p* < .001, η^2^ = .001, and recency recall *F*(2,15166)=8.67, *p* < .001, η^2^ = .001. Specifically, the SCD+worry group showed lower primacy recall than controls (*p* < .001), whereas recency recall was lower in controls than both the SCD+worry (*p* < .001) and SCD‐no worry groups (*p* = 0.02). For delayed recall, a main effect of SCD status was found on both total scores, *F*(2,15166)=3.74, *p* = 0.02, η^2^< .001, and primacy recall, *F*(2,15166)=4.32, *p* = 0.01, η^2^ = .001, with the SCD+worry group performing worse than controls (*p* = 0.02 and *p* = 0.01, respectively). No differences were observed for delayed recency recall.

**Conclusion:**

Older adults with SCD and related worry consistently showed lower primacy recall than controls, suggesting that SPE patterns may potentially differentiate individuals with SCD who are at‐risk of developing AD. As such, evaluation of SPE patterns may further improve early detection of cognitive decline in this population.

